# Low use of data analytics for health sector priority-setting in Ghana: A case for strengthening analytical capacity

**DOI:** 10.1371/journal.pgph.0004981

**Published:** 2026-03-03

**Authors:** Emmanuelle A. Dankwa, Catherine Wambura, Marwatunnisa Al Mubarokah, Christina J. Matta, Bellama Gado, Joyce Komesuor, John Van Savage II, Anna Makido, Frank Baiden, Abdisalan M. Noor

**Affiliations:** 1 Department of Global Health and Population, Harvard T.H. Chan School of Public Health, Boston, Massachusetts, United States of America; 2 Department of Immunology and Infectious Diseases, Harvard T.H. Chan School of Public Health, Boston, Massachusetts, United States of America; 3 Fred N Binka School of Public Health, University of Health and Allied Sciences, Hohoe, Volta Region, Ghana; 4 Applied Health Analytics for Delivery and Innovation (AHADI), Nairobi, Kenya; University of Essex, UNITED KINGDOM OF GREAT BRITAIN AND NORTHERN IRELAND

## Abstract

Ghana has implemented health sector reforms to improve health care access in alignment with Sustainable Development Goal 3. However, Ghana’s health sector faces challenges including persistent overspending of the healthcare budget. These challenges are exacerbated by recent cuts in external aid to Ghana, illustrating the importance of effective health sector priority-setting (HSPS)—the process of determining how to allocate resources best to maximize population health outcomes among various alternative, competing health needs and interventions. Although Ghana has a history of HSPS initiatives, there is no authoritative source on the current landscape of HSPS in Ghana and on the use of data and analytics. Combining key informant interviews with an extensive literature review, this study aimed to provide an overview of current HSPS processes at the national level in Ghana, focusing on the use of data and analytics. Eight interviewees were selected from governmental health institutions, development partner organizations and academia through a combination of purposive and snowball sampling. Findings show some good practices regarding the use of data and analytics for HSPS in Ghana, including the existence of a routine health database for Ghana Health Service (GHS) facilities, existence of training programs on data collection for GHS staff and some use of subnational data for national-level decision making. However, many interviews showed that the use of data and analytics for HSPS was low mainly due to low analytical capacity within the GHS and MoH, donor and political influences on the process, and a fragmented health data system. Interviewees recommended the following to increase the use of data and analytics in HSPS: 1) increase training in data analytics and data-driven decision-making, particularly among senior-level staff in the GHS and Ministry of Health, 2) establish a centralized health research coordination agency, and 3) integrate and coordinate health databases.

## 1. Introduction

Ghana has passed policies and reforms to improve healthcare access and establish a health system based on the Universal Health Coverage (UHC) principles. Since the 1990s, Ghana has implemented reforms within its health sector, including adopting decentralized governance and collaborative frameworks that engage various levels of government, civil society organizations (CSOs), the private sector, and international organizations. These reforms aimed to enhance service delivery at the community level while addressing the health needs across Ghana’s urban and rural areas [1,2].

Ghana allocates 2.02% of its GDP to healthcare, and this is channeled mainly through its Ministry of Health (MoH) [3]. The health funding structure relies on multiple sources, with the government of Ghana being the primary contributor at 54% of the budget in 2023, and development partner funding contributing 20% of the budget in that year [3,4]. A key challenge of healthcare spending in Ghana is the overspending of the healthcare budget in recent years: between 2019 and 2022, the MoH expenditure exceeded the budget by 6%-13% [3], demonstrating that there are more healthcare expenses than allowed by the budget. A related challenge is the underfunding of the budget, resulting in important health needs being unmet.

Given these challenges and with the recent cuts in external aid to Ghana [5], health sector priority-setting (HSPS) is critical for the Ghanaian context. HSPS is the process of determining how to efficiently and effectively allocate limited resources to meet population health needs. It is the third stage of a country’s health policy and planning processes which typically consists of eight stages: population consultation, health situational analysis, HSPS, national health strategic planning, operational planning, costing, budgeting, and monitoring and evaluation [6].

Ghana has a history of priority-setting initiatives, including the International Decision Support Initiative (iDSI) [7] and the recent launch of the first strategy for Health Technology Assessment (HTA) [8,9]. HTA, recommended by the WHO to be used by member states, describes standard processes to evaluate the value (effectiveness, cost-effectiveness and consequences) of a health technology or intervention [10]. Despite these emerging initiatives and the relevance of HSPS for Ghana’s context, there is no authoritative source on the current landscape of HSPS in Ghana and particularly on the use of data and analytics in the process. References on the subject either focus on a single HSPS initiative [7,9,11] or priority-setting for a single aspect of the health sector [12]. No source, as far as we know, explains the HSPS process at the national level and the role of data and analytics in the processes. This presents a gap in understanding the use of data and analytics for HSPS in Ghana, making it challenging to propose any related recommendations for improvement.

This paper sought to address this gap in the literature. Combining key informant interviews (KIIs) with an extensive literature review, the paper offers an overview of current HSPS processes at the national level in Ghana, focusing on the use of data and analytics in HSPS. By “use of data,” we mean the translation of data into insights for health decision making routinely for operational purposes, strategic development and policy change. Reporting of raw data is not considered as data use, even if it is a core function without which data use is limited. This process of translation typically involves the application of analytic methods to the data. This process of translation typically involves the application of analytic methods to the data. The paper is outlined as follows: 1) The methods are discussed. 2) Then, to establish context, the health governance and health delivery system of Ghana and priority-setting processes are presented based on the literature review. 3) Results from the interviews are then presented, organized by three themes: HSPS processes; use of data and analytics in these processes; and successes and challenges associated with the use of data and analytics for HSPS. 4) Recommendations for increased use of data and analytics for HSPS are also suggested, based on the interviews. 5) Finally, a discussion summarizing key points of the paper is presented.

## 2. Methods

The study used data from two complementing sources: a literature review and KIIs. The KIIs were intended to fill in relevant information gaps left by the extensive literature review.

### 2.1 Ethical review

Ethical approval for this study was not required by the Institutional Review Board (IRB) of the Harvard T.H. Chan School of Public Health due to the determination that the study did not constitute human subjects research as defined by Department of Health and Human Services regulations or Food and Drug Administration regulations. The IRB provided a determination letter to confirm this research qualifies as non-human subject research (Protocol number: IRB24–0942). Participants provided written informed consent to participate.

### 2.2 Literature review

The literature review included peer-reviewed research articles, grey literature, and essential policy documents to explore Ghana’s HSPS processes. This desk review examined crucial aspects, such as HSPS implementation steps, the roles of involved individuals and institutions and data and analytics usage in the process. The search for relevant sources covered prominent public health databases, including Elsevier, PubMed, and Google Scholar, focusing on documents published within the past decade to ensure up-to-date insights. Additional information was obtained from the MoH and Ghana Health Service (GHS) websites focusing on health governance and how the different agencies and departments are involved in the HSPS processes.

### 2.3 Qualitative study

#### 2.3.1 Study design.

A case study design was used for this study. The qualitative study involved KIIs and used a semi-structured interview approach. Questions were structured to fill the gaps identified in the literature review. Interview guides are available in S2 Text. The style and content of questions were adapted to suit the background, expertise, and institution of participants. The semi-structured approach allowed for the flexibility for follow-up questions to explore any relevant themes which arose during interviews if not captured in the prepared questions. KIIs were conducted in August 2024 in Accra, Ghana with individuals directly or indirectly involved in HSPS to gather contemporary insights on HSPS practices in Ghana.

#### 2.3.2 Study population, recruitment, and sampling.

Eight participants were selected through a combination of purposive and snowball sampling from governmental institutions, academia, and partner organizations. This enabled a targeted selection of participants who are well-versed in the health sector in Ghana, most of whom had worked within their organizations and roles at senior levels, allowing for participants to paint a robust picture of Ghana’s priority-setting processes. Participants were current or former employees of two governmental agencies (MoH and GHS), two development partner or donor organizations (World Health Organization (WHO) and Program for Appropriate Technology in Health (PATH)), and one academic institution experienced in collaborating with the GHS (University of Health and Allied Sciences, Ghana). Additional participants were not sought after saturation had been achieved by the eighth interview. Saturation was determined by observing that interview summary notes for the last few interviews showed no new major themes, suggesting that additional data collection was unlikely to result in additional findings [13,14].

#### 2.3.3 Data collection.

Interviews were conducted separately for each participant by two to four interviewers. Interviews were in English and were conducted in-person or online using Zoom, according to participants’ preferences. Five interviews were held via Zoom while three interviews were in person.

Participants’ voices were not recorded due to the concern that with recording, some participants may not feel comfortable to freely respond to some of the questions since they bordered on sensitive issues including political and leadership issues. Interviews were therefore recorded through notetaking by two researchers and were summarized by one researcher who compared notes to ensure consistency. Summary involved paraphrasing participants’ responses while retaining relevant details. To ensure accuracy, prior to being analyzed, the summarized notes were again reviewed by one researcher who was present at all interviews.

#### 2.3.4 Data analysis.

The analysis was conducted collaboratively using a deductive-inductive thematic analysis approach [15]. The pre-defined categories were developed by two researchers based on the literature review that was done prior to qualitative analysis (deductive approach). These categories were further refined during the initial familiarization process, in which both researchers read a subset of interview summary notes with purposeful overlap to facilitate discussion and consensus.

In parallel, open coding was also conducted to capture emergent ideas and themes directly from the interviews (inductive component). Codes were iteratively compared, collapsed, and refined. Each interview was then independently coded by two researchers, followed by a comparison of codes and discussions to resolve any discrepancies. NVivo 14 software was used throughout the analysis process, including to explore and organize the data, for coding and theme development, to visualize connections between emerging themes, and examine the patterns both within individual codes and across the entire dataset. These two researchers also used NVivo14 to compare and discuss inter-rater consistency, such as keeping short memos for questions or suggestions.

To enhance rigor, additional examination of themes and codes was conducted by two other researchers on the team to ensure inter-coder reliability for consistent coding. This iterative, collaborative analysis process allowed each team member to analyze specific codes and themes, with analysis interpretations regularly discussed within the research team to refine interpretations.

#### 2.3.5 Reflexivity statement.

The research was conducted collaboratively using a reflexive approach, with a diverse team of various races, ethnicities, and nationalities, who brought varying backgrounds, expertise, and personal experiences. This provided a balanced foundation for analyzing complex processes and allowed for an examination of the priority-setting processes through multiple perspectives, adding depth and breadth to our findings. The stakeholder consultations were conducted in English, the primary language in Ghana, facilitating effective communication with participants. KIIs were led by researchers and native Ghanaians at the University of Health and Allied Sciences which helped capture subtle nuances and foster trust with key informants. Analysis was led by researchers at the Harvard T.H. Chan School of Public Health and validated by University of Health and Allied Sciences colleagues. The team’s collective expertise in literature review, qualitative methodologies, and health systems strengthened the study’s rigor and systematic approach. Despite efforts to ensure objectivity, the researchers are aware that inherent biases may subtly influence the interpretation of the findings. Transparency, therefore, is prioritized in presenting conclusions, with recognition that the research team’s backgrounds may shape perspectives and analysis.

#### 2.3.6 Inclusivity in global research.

Additional information regarding the ethical, cultural, and scientific considerations specific to inclusivity in global research is included in the Supporting Information (S1 Checklist).

## 3. Information from the literature

### 3.1 Health governance structure

The MoH is the primary policymaking and regulatory body responsible for central-level activities, including policymaking, regulation, and planning coordination [16]. Authority from the Ministry of Health is delegated to its agencies, including the Ghana Health Service (GHS), Teaching Hospitals, and faith-based service providers. The GHS is the main implementation arm on government healthcare policies and administrates healthcare delivery, controlling the operations of most government health care facilities. The GHS comprises 11 divisions, including the Public Health Division, the Research and Development Division (RDD) and the Policy, Planning, Monitoring, and Evaluation Division (PPME) (Fig A in S1 Text). Each division is headed by a divisional director who reports directly to the Director General. The Public Health Division oversees disease-specific control programs, such as the Tuberculosis (TB) Control Program, which are headed by program managers.

### 3.2 Health delivery system

The health delivery system is organized into three tiers (Fig B in S1 Text). The primary level includes district hospitals, sub-district health centers, and community-based health planning services [16]. This level aims to provide community members with basic essential primary health care services, which are critical for the attainment of UHC. The secondary level, represented by regional hospitals, offers public health and clinical services and acts as referral centers for primary level facilities. At the tertiary level, specialized care delivery is provided through teaching, university, and psychiatry hospitals which serve as hubs for training health professionals and provide services to clients referred from the other two levels [16]. At the primary and secondary levels, health directorates manage health service delivery [17].

The private sector and CSOs also support the government’s efforts in healthcare service delivery, accounting for 19% of outpatient department visits, with facilities predominantly located in urban and peri-urban areas [16]. Faith-based organizations also support governmental efforts to provide primary and secondary level care by establishing and operating health facilities [16].

### 3.3 Health service data

Ghana has several health data sources, such as the Demographic and Health Survey (DHS) data, Global Burden of Disease, National Health Insurance Scheme data, Lightwave Health Information Management System (LHIMS), and the District Health Information Management System II (DHIMS2) which is the main repository of health service data for the GHS and MoH [9,18]. All health sector data in Ghana is managed by the Centre for Health Information Management under the Information Monitoring and Evaluation Department of the GHS’ PPME [9]. However, there is no composite database for all healthcare data and existing systems have limited interoperability; therefore, data remains largely compartmentalized [9].

For details on the structure, function and challenges associated with major health data sources in Ghana, see S1 Text.

#### 3.3.1 Data collection within DHIMS2.

The DHIMS2 is a nationwide, web-based health information platform managed by the GHS. At the GHS facility level, patient encounters and service delivery indicators are initially recorded in standardized paper registers during routine clinical care. These data are subsequently aggregated into monthly summary reports and entered electronically into DHIMS2 at the sub-district or district level, where they are validated and transmitted to the regional and national levels to be aggregated within DHIMS2 [19].

### 3.4 Priority-setting processes

#### 3.4.1 Overview.

Ghana’s priority-setting process is rooted in a constitutional mandate spearheaded by the National Development Planning Commission, which guides public and civil society organizations in shaping strategic plans [16]. The Health Sector Medium-Term Development Plan (HSMTDP) is the blueprint for Ghana’s health system, guiding investments, interventions and reforms.

The 2022–2025 HSMTDP outlines the health sector’s priorities for the period, which were determined based on the following [16]: 1) a review of the previous (2018–2021) HSTMDP, 2) a holistic assessment of the health sector conducted in 2020 [20], and 3) the *aide memoire*, a document summarizing priority decisions made by government and development partners at the annual health sector summit. The development of the 2022–2025 HSMTDP was also guided by the Ghana National Health Policy [2] and the UHC Roadmap for Ghana (2020–2030). An annual Program of Work (PoW) document outlines the action plan for implementation of the HSMTDP for each year. For example, the 2024 PoW [21] outlines priority areas for focus in 2024, aligning with the 2022–2025 HSMTDP.

#### 3.4.2 Health system assessment.

The MoH conducts a performance assessment of interventions and health activities implemented according to the previous strategic plan and uses the assessments inform priorities for a new plan. In addition to a national assessment of the health sector (known as the Holistic Assessment), area-specific assessments, such as on public expenditure and public health emergencies, are conducted [22]. For example, a review of the 2018–2021 HSMTDP informed priorities for the 2022–2025 HSMTDP. The MoH collaborates with its agencies, development partners and other health stakeholders in the development of these priorities [16].

#### 3.4.3 The health summit.

Findings on the assessments of the implementation of the previous health strategic plan are presented to health sector stakeholders at an annual health sector planning meeting known as the annual health summit, hosted by the MoH [22,23]. For example, the 2024 summit presented results from the mid-term review of the HSMTDP, providing an opportunity for the MoH, GHS, and other invited health sector stakeholders to discuss the report and share insights into the health system’s performance and effectiveness based on stated policy objectives outlined in the HSMTDP and the UHC Roadmap of 2020 – 2030 [24]. Within the health summit, a “business meeting” is held to finalize priorities. This meeting is held between the management of the MoH, directors of MoH agencies, and development partners [24]. Priorities finalized in this meeting are summarized into the *aide memoire*, which informs the PoW for the upcoming year [8].

#### 3.4.4 Inter-agency leadership committee.

Another important avenue for HSPS is a regular meeting between key health sector leaders in government, known as the Inter-Agency Leadership Committee. It exists to facilitate unity in the health sector by providing a platform for the exchange of ideas which will “influence policy and provide overall strategic direction in the health sector” [22]. It meets at least quarterly and comprises the Ministers of Health, Chief Director of the MoH, heads of MoH agencies, Director General of the Ghana Aids Commission (the Commission is under the Office of the President and is not an agency of the MoH) and the PPME Director, who is the committee’s secretary. It is chaired by the Minister of Health. Other stakeholders external to the government may be invited to the Committee’s meetings as needed.

#### 3.4.5 Health sector working group.

The Health Sector Working Group is a forum for engagement between government health sector leaders and sector partners external to the government, including civil society organizations, local and international non-governmental organizations, development partners and the private sector [22]. The Working Group exists to facilitate policy dialogues among key sector partners, monitor programs such that they achieve desired aims, improve the alignment of development partners’ activities with government priorities, review resource allocation and discuss and agree on the annual PoW [22]. These meetings are held quarterly and chaired by the Minister of Health.

#### 3.4.6 Examples of data to decision pathways in Ghana.

***Maternal health: From data to priority*:** The country’s capacity to set data-driven health priorities has evolved at both the national and program-specific levels, enabling health facilities to contribute essential evidence for the development of meaningful policies. For example, assessment of reproductive and maternal health indicators in the DHIMS2 revealed low coverage of skilled birthing attendance at delivery as a challenge to reducing maternal and neonatal mortality, coupled with shortages of the number of midwives to meet the needs of the population of women of reproductive age. In response to this data, and to address the Millenium Development Goal of universal skilled attendance at delivery, in 2003, the GHS and the Ghanaian government made a key policy change introducing a direct training program for midwives, no longer requiring a nursing background to attend a specialized midwifery program [25]. This change resulted in improvement of the percentage of births with a skilled attendance at delivery (47.1% in 2003 to 58.7% in 2008) [26], and reductions in the maternal mortality ratio (204.5 per 100,000 live births in 2003 compared to 199.7 per 100,000 live births in 2008) [27].

***Establishment of National Health Insurance Scheme: From data to priority*:** Prior to 2003, payment for healthcare in Ghana was based on a “cash and carry” system, where patients paid fully for healthcare. However, several issues were identified with this system, as spelt out in the *National Health Insurance Policy Framework* of the Ministry of Health [28]. These include declining use of health services due to cost barriers and evidence that ~80% of people needing care could not afford it at the time of service. These challenges precipitated the establishment of a National Health Insurance Scheme (NHIS). Data from the seventh round of the Ghana Living Standards Survey conducted in 2016/2017 show a 26% increase in healthcare utilization and a 4% decrease in out-of-pocket payments for individuals enrolled in the NHIS [29], demonstrating that the establishment of the scheme reduces health care access barriers.

***Introduction of Community-Based Health Planning and Services: From data to priority*:** In the 1990s, health system data in rural Ghana revealed persistent geographic barriers to basic primary care, with maternal and child health outcomes remaining poor in remote districts [30]. To generate evidence on how community-level delivery could improve these outcomes, the Navrongo Health Research Centre implemented the Navrongo Community Health and Family Planning Project (1994–2003)––a field experiment testing the deployment of trained nurses (community health officers) into community settings to deliver preventive, maternal, child, and basic curative services directly in communities rather than only at distant facilities. Data from this trial showed significant reductions in mortality, with under-five mortality declining by 43.6% and infant mortality declining by 34.5% between 1995 and 2003 in the Navrongo study area compared to comparison zones [31]. Based on these findings, the Government of Ghana adopted the Navrongo model nationally in 1999 as the Community-Based Health Planning and Services (CHPS) initiative. An extensive review of CHPS evaluations show the policy to be effective in reducing under-five mortality, enhancing family planning, and improving the uptake in maternal and child health services, with a 56% increase in the odds of skilled birth attendant care [32].

### 3.5 Gaps in the literature

Regarding the health system assessments which precede and influence priority-setting, the literature does not have information on how exactly these assessments are conducted, the data sources and analytics used to perform these assessments, the agencies which provide data for these assessments, and how district or regional level assessments inform national assessments. The literature does not discuss successes or challenges associated with data and analytics for HSPS in Ghana. Consequently, there are no recommendations for the improvement of the use of data and analytics for HSPS in Ghana. The interviews and qualitative analysis presented in the next section seek to address these gaps.

## 4. Results from qualitative analysis

Three relevant themes were identified from the KIIs: 1) priority-setting processes (from data to priorities), 2) data and analytics for HSPS and 3) successes and challenges in the use of data and analytics for HSPS. These are now discussed by theme (Table 1). Interview notes are available in S3 Text.

**Table 1:** Themes identified from qualitative key information interviews.

Themes	Sub-themes
Priority-setting processes	• Health system assessments • Processes involved from assessments to priorities
Data and analytics	• Data analysis processes • Data and analytic capacity
Successes and challenges associated with the use of data and analytics	• Successes • Challenges

### 4.1 Theme 1: Priority-setting processes

#### 4.1.1 Health system assessments and reviews.

***Literature gap*:** The literature does not explain how assessments are conducted within the GHS and other MoH agencies; in particular, the coordination between GHS divisions, across GHS governance levels (district, regional, national) and between the GHS and development partners.

***What this study adds*:**

**Preparation of GHS assessments:** To prepare the assessment report by the GHS, each GHS division and program at the national level conducts an annual performance review (APR) of its activities and prepares an assessment report [Interviews 3, 5]. These APRs assess performance indicators against priorities [Interview 5] as outlined in the PoW for the previous year [Interview 2]. Data for these assessments are mainly sourced from DHIMS2 [Interview 4], though demographic data are also used [Interview 2]. Divisional directors present and discuss their reports in a meeting chaired by the Director General of the GHS [Interview 3]. At the district and regional levels of health service delivery, health directorates also conduct thorough APRs [Interview 5], in addition to quarterly activity assessments [9]. District assessments are collated at the regional level and regional assessments are collated at the national level [Interview 5]. Aside these assessments, as a monitoring strategy, the PPME also conducts a quarterly assessment of health services based on DHIMS2 data [Interview 5]. These assessments (district, regional, divisional and PPME) are collated by the PPME into an overall GHS assessment [Interviews 3, 5]. Therefore, the national GHS assessment is a composite of divisional and sub-national assessments.

**Health indicators used in assessments:** The health indicators considered in an analysis are dependent on the objectives of an assessment or the requester; for example, prior to certain meetings, the GHS Director may request analyses on health indicators which are relevant to the meeting theme [Interview 2]. Good coordination between the PPME and the MoH enables the MoH to present routine, up-to-date data in specific meetings while also allowing the PPME to stay informed of the MoH’s priorities and targets [Interview 2].

#### 4.1.2 From assessments to priorities.

***Literature gap*:** The literature is not clear on the processes which occur between the final assessment and the final set of priorities.

***What this study adds*:** The process by which assessments influence priorities was explained as follows.

**MoH agencies’ meeting:** The GHS Director General decides which aspects of the GHS assessment to highlight at a meeting chaired by the Minister of Health and attended by other MOH agency heads (e.g., heads of teaching hospitals) who also report on their agency assessments [Interview 3]. As the GHS assessment is a composite of the divisional and subnational assessments, data and priorities outlined in quarterly reviews at the subnational level are expected to inform priority areas [Interviews 3, 5]. Decisions by the Director General of the GHS on what to highlight at the MoH meeting are generally based on the burden of the health problem as showed by the assessments [Interview 3] or on emerging health needs [Interview 5].

After assessments from the GHS and other MoH agencies are presented to the Minister of Health, an ad hoc committee led by the PPME director is formed to synthesize these assessments into an assessment of the national health situation which contributes to the *aide memoire* [Interview 4]. The constitution of the committee depends on the health indicators or issues that were highlighted for attention, based on presentations by agencies [Interview 4]. The Minister of Health presents this assessment to government leaders [Interview 3].

**Priority scoring at health summit:** During the annual health summit, all agencies of the MoH, including the GHS, are expected to report on the assessments of their PoW for the previous year [Interview 2]. Attendance to the last day of the summit, referred to as the business meeting, is limited to the minister, relevant agencies, select staff from the GHS and the MoH at the national level and development partners, including PATH [Interviews 1, 5]. This meeting serves as a deliberative process to assign scores to various priorities, which ultimately determines a set of priorities based on the highest score across all indicators [Interviews 1, 7]. There was no evidence from the interviews that any clear criteria or weights were used in the scoring process or in the achievement of consensus. Additionally, as participation in the business meeting is limited to politicians and only a few technocrats at the national level, actors involved in the deliberation process is influenced by rank rather than analytic capacity [Interview 5]. The *aide memoire* outlines the agreed priorities and funding commitments between the government and partners [Interview 4, 5] for the subsequent year. Some development partners such as PATH do not directly influence priority decisions at the health summit but rather do so at the regional and national levels [Interview 1].

**Factors determining priorities:** Several factors shape the determination of priorities. These include the seriousness of the health problem assessed by the associated burden of disease; resource or funding availability; emerging health trends; and the global health agenda, particularly the Sustainable Development Goal 3 and the UHC roadmap [Interviews 1, 4, 5, 8]. The latter factor is because Ghana is keen to remain connected to the global agenda and related international programs [Interview 2]. In terms of availability of resources, for instance, out of the funds allocated to the health sector, close to 70% goes to personal emoluments, leaving only 30% for activities within the ministry, which is insufficient [Interview 2].

Additionally, donors usually have their own priorities; for example, USAID prioritized health system strengthening, monitoring, and evaluation, while the World Bank prioritizes programs such as HIV and TB [Interviews 2, 5]. PATH also has priority focus areas but regularly conducts its own situational analysis to identify gaps and uses this information to determine priority areas requiring its support [Interview 1]. As an additional example, an interviewee noted that in the past, one funder only supported work in five (out of ten) regions because the funder had designated those as its priority regions, due to poverty levels there [Interview 5].

Political influences on the priority-setting process further exacerbate this issue of misalignment of priorities and country needs [Interviews 1, 5]. Health priorities have sometimes been revised to incorporate manifesto promises, even if that did not reflect prevailing needs. For example, during the 2018 annual health summit, the Vice President mentioned the use of drones to deliver vaccines which was not a health sector priority then, but policies were later developed to accommodate the Vice President’s comments [Interview 5].

#### 4.1.3 Summary of HSPS processes.

**Fig 1:**
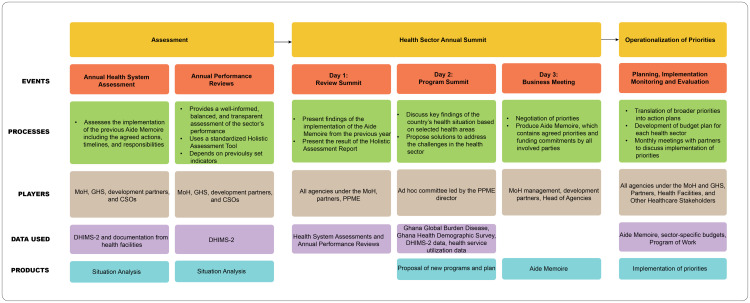
Ghana’s step-by-step priority-setting processes.

The step-by-step process of priority setting at the national level in Ghana, as explained by the key informants and from the literature, is summarized in Fig 1. The figure accounts for the events, processes, players, data used and products at each stage of HSPS in Ghana at the national level.

### 4.2 Theme 3: Data and analytics used in priority-setting

#### 4.2.1 Data analysis processes.

***Literature gap*:** Although the literature provides information on the data sources that are used in priority-setting, the processes and methods used in analyzing the data are not discussed.

***What this study adds*:**

**Data management and pre-analysis processes:** GHS uses the routine surveillance data in DHIMS2 for prioritization [Interviews 2, 5, 8]. Examples of aggregated data which inform priorities include disease burden data, health service utilization data, and demographics [Interviews 2, 3].

PPME is responsible for managing and analyzing the data in DHIMS2 [Interviews 2, 4]. Before analyzing the data, PPME checks its accuracy, timeliness, and completeness by verifying with facilities or health directorates at multiple levels [Interview 2]. Within the health directorates, data management is led by health information officers who represent the PPME at their levels [Interview 2]. The RDD of the GHS also conducts research to inform GHS priorities [Interview 3].

**Analytic methods used:** After validation, the data is analyzed in some form, using methods such as trend analysis, spot mapping using GIS, graphs, or pie charts [Interview 2]. Generally, there is a reliance on basic descriptive analytics such as counts, averages, proportions and frequencies, and a lack of usage of more critical or advanced analytics such as analysis of relationships between factors [Interviews 3, 4], predictive or prescriptive analysis [Interview 2], big data analysis and data visualization [Interview 4].

**Other institutions aside GHS contributing to analytics:** Local academic institutions including Kwame Nkrumah University of Science and Technology, University of Ghana School of Public Health and University of Health and Allied Sciences, in partnership with the GHS, also conduct research to support priority-setting [Interviews 2, 6]. Given that DHIMS2 is not publicly accessible, researchers external to the GHS and commercial agencies need to request access to the database [Interviews 2, 3].

#### 4.2.2 Data and analytic capacity.

***Literature gap*:** No literature source examines the local data and analytic capacity for HSPS in Ghana. Specifically, information is absent on the gaps in local capacity, on efforts aimed at addressing these gaps, and the areas these efforts are focused.

***What this study adds*:**

1. **State of analytic capacity:** Despite the richness of data that the Government of Ghana has, its capacity to utilize and analyze the data and transform analysis results into action through policy is still very limited [Interviews 3, 4, 5, 7]. Multiple interviewees emphasized that the issue is not a lack of data; rather, the main challenge lies in capacity to synthesize, analyze, and translate the data into actionable policy [Interviews 2, 4, 7]. Understanding of the results of advanced analytics among decision makers is also weak [Interviews 2, 4]. As a result of these gaps in capacity, deeper insights from data, such as on cost-effectiveness of an intervention, are rarely available to inform decision-making [Interviews 2, 4].
2. **Efforts to improve analytic capacity:** To address these challenges, several international agencies have been assisting Ghana in improving skills across the data life cycle. In data collection, PATH experts help to identify gaps and support the MoH to strengthen the government’s data system by improving data capture tools; for example, supporting with the addition of health indicators to the system [Interview 1]. Additionally, the WHO, as one of Ghana’s primary international partners, plays a key role in supporting Ghana’s use of the SCORE [33] assessment [Interview 7] and conducting district-level health facility assessments [Interview 7]. WHO support extends to specialized areas, such as capacity building within the tuberculosis program to improve modeling for TB incidence estimates, thereby improving the country’s disease burden assessments [Interview 7]. The WHO also conducts training for government officials and MoH stakeholders on data analysis, emphasizing the use of data for policy briefs and infographics [Interview 7]. This is intended to bolster their ability to interpret analysis results, identify programmatic gaps, and promote data-driven decision-making. Faculty in academic institutions also receive training from the WHO in implementation research to align their work more closely with national policy needs, strengthening the role of academic institutions in evidence-based policymaking [Interview 6].

Other international agencies also support capacity-building efforts, with the flexibility to determine the specific level of capacity they aim to strengthen [Interview 2]. However, at the national level, the PPME division determines the training content [Interview 2]. Various capacity-building trainings have been conducted in 2024, such as trainings on DHIMS2 data quality, data management, standard operation procedure, district health system functionality assessment, harmonized health facility assessment, and health equity assessment [Interviews 2, 7]. By overseeing training content, PPME ensures alignment with the skills most needed by staff. For instance, DHS training specifically trains on using access mode and Quantum Geographic Information System (QGIS) for improving accessibility of emergency referrals from community-based health planning services [Interview 7].

Through capacity-building, the Information and Communication Technology department of the PPME ensures that information officers at various levels can utilize data collection platforms optimally [Interview 2]. The department provides e-learning programs that have reached over 14,000 health sector staff within six years of implementation, covering various skills such as training in digital tools and descriptive analysis [Interview 2]. Training is also conducted when new features are added to DHIMS2, to ensure the quality of data entered [Interview 2]. There are efforts to train staff in predictive and prescriptive analysis [Interview 2].

### 4.3 Theme 4: Successes and challenges associated with the use of data and analytics for HSPS

**Fig 2:**
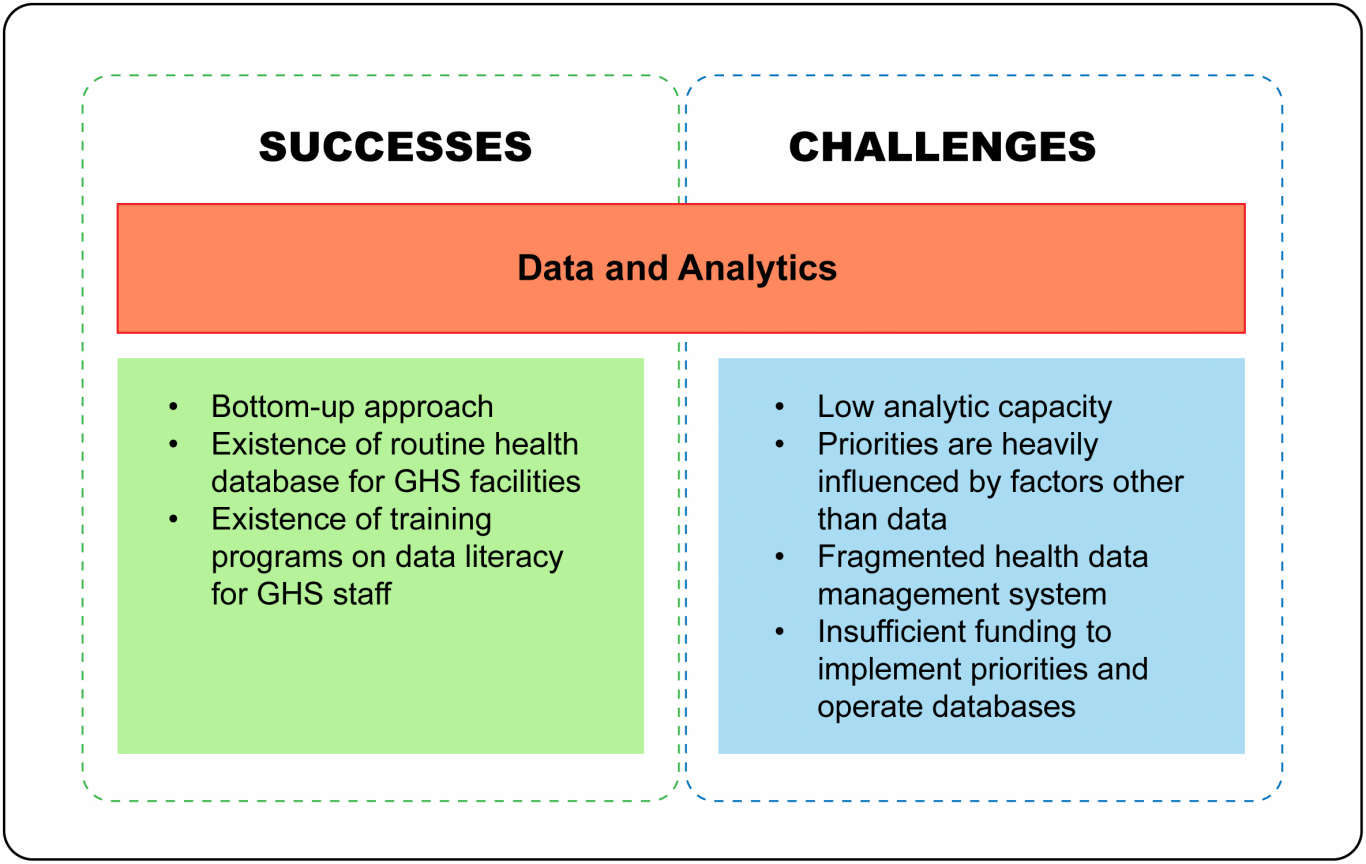
Summary of successes and challenges associated with the use of data and analytics for health sector priority-setting in Ghana. GHS = Ghana Health Service.

Successes and challenges associated with the use of data and analytics for HSPS in Ghana are now discussed and summarized in Fig 2.

#### 4.3.1 Successes.

1. **Bottom-up approach:** The health assessments conducted at the sub-national level, based on sub-national data, constitute the national assessment [Interviews 1, 5, 7]. This bottom-up approach is important for HSPS in Ghana [Interviews 1], ensuring that local needs inform national strategies. In 2016/2017, Ghana introduced tablets for data collection at the community level [Interview 5], enabling seamless transfer of data to the national level so that local insights shape national health policies in a timely fashion.
2. **Existence of routine health database for GHS facilities:** DHIMS2, the main data repository for GHS facilities, consolidates information from routine health activities, creating a resource for decision-making [Interviews 2, 3, 4, 5, 6].
3. **Existence of training programs on data literacy for GHS staff:** Ghana has also invested in its health workforce by implementing an e-training program. Over the past six years, more than 14,000 health workers have participated in the program, learning how to collect and analyze data with precision [Interview 2]. There is also support from development partners such as WHO [Interview 7] and PATH [Interview 1] to implement similar training programs.

#### 4.3.2 Challenges.

1. **Low analytic capacity:** A lack of demand for advanced analytics (e.g., assessing relationships as opposed to simply observing counts and averages), a shortage of highly skilled professionals in data analytics, and high staff turnover within the GHS and MoH exacerbates the analytic capacity gap [Interview 3, 4] contribute to low data analytic capacity. This lack of demand for advanced analytics creates a disincentive for current GHS staff to pursue further training in advanced data analysis and for doctoral level data analysts to work with the GHS [Interview 3].
2. **Priorities are heavily influenced by factors other than data:** While donor funding has been instrumental in supporting various health initiatives, it often comes with specific requirements or preferences. Donors may prioritize certain diseases or interventions that meet their organizational objectives or reflect international health trends but do not align with national health priorities determined based on data [Interviews 2, 3, 5, 8]. However, development partner representatives from WHO and PATH stated that their activities align with national priorities [Interviews 1, 7]. The WHO representative also stated that its priorities are based on country data [Interview 7]. They explained that there are usually no tensions between WHO and the government with respect to what the priorities should be; however, the order of priorities usually requires discussions before agreement is reached between the parties [Interview 7].

Political interference also influences HSPS, as decisions are sometimes swayed by shifting political agendas rather than the health needs of the population [Interviews 1, 3, 5].

1. **Fragmented health data management system:** Although the Health Facilities Regulatory Agency (HEFRA) mandates all health facilities to report health service data to the DHIMS2, teaching hospitals and some private facilities do not report their data [Interviews 2, 3, 4]. Given that these systems are not integrated [9], this situation creates a fragmented health data management system, limiting the opportunities to effectively use this data for setting priorities that address health issues nationally. As of August 2024, about 400 health facilities were not reporting to the DHIMS2 [Interview 4].
2. **Insufficient funding to implement priorities and operate databases:** Insufficient funding to implement identified priorities could be a demotivating factor for conducting data analytics for priority setting [Interview 7]. Funding for implementation is typically limited or allocated only to specific areas; therefore, priorities without dedicated financial backing are often delayed or abandoned [Interviews 3, 5, 7]. Requested funds for project implementation are seldom available to the GHS because the Ministry of Finance, in charge of disbursing health sector funds, usually prioritizes staff salaries [Interview 5]. These make up about 70% of the funds allocated to the health sector, leaving only about 30%—an insufficient fraction—for project implementation across over 23 MoH agencies [Interview 2]. The TB Control Program within the GHS, for example, is underfunded by 45% [Interview 8]. This means that some health programs, although identified as a priority, may never be implemented if they do not attract donor interest or government allocation, limiting the impact of Ghana’s strong evidence-based planning frameworks. Indeed, an interviewee noted that research findings are often left on the shelves and inform policies when it has a lot of funding to back it [Interview 3].

Limited funding also hinders the operability of health databases in several aspects. Without funding for good internet connectivity, the ability to use the DHIMS or e-Tracker is limited as these databases are online [Interview 2]. Financial constraints have also prevented the full migration from the paper system to the electronic system for data management, leading to an increased workload for staff who need to operate both systems [Interview 2]. For example, of the 2000 Directly Observed Therapy centers for TB treatment, only 425 facilities—less than a quarter—report their data electronically as of August 2024 [Interview 8]. Insufficient funding also limits the feasibility of conducting routine research on the HIS, which is vital for its quality improvement [Interview 2]. Research with the HIS data is also insufficiently funded. Therefore, evidence suited to the local context is not always available, causing stakeholders to adopt policies based on evidence from other countries [Interview 3, 4]. Such policies are likely to fail, given that context-specific information impacts generated evidence.

### 4.4 Recommendations for increased use of data and analytics for HSPS

**Fig 3:**
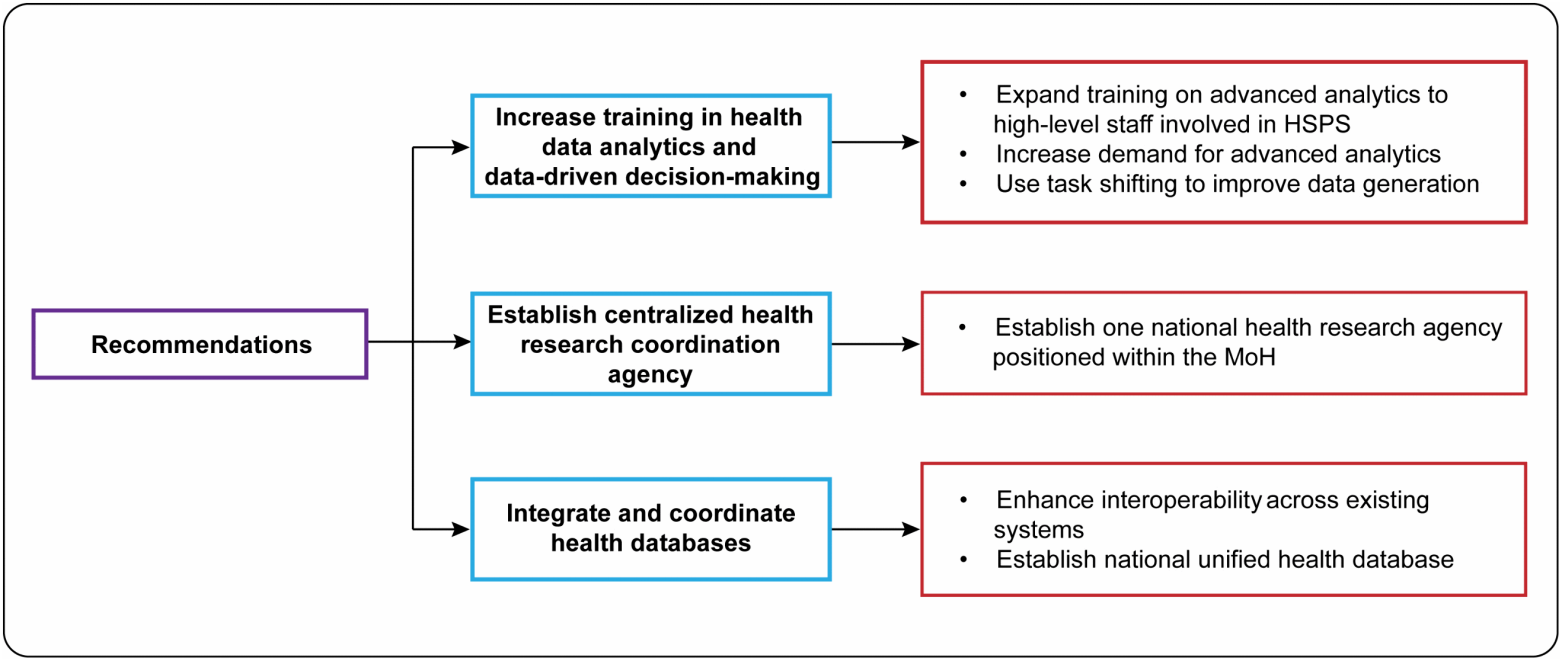
Recommendations for increased use of data and analytics for health sector priority-setting in Ghana. HSPS = Health sector priority-setting. MoH = Ministry of Health.

The following recommendations outline actions for improvement in the use of analytics for HSPS in Ghana. These recommendations are formulated based on information from the interviews. These recommendations are summarized in Fig 3 and detailed below.

1. **Increase training in data analytics and data-driven decision-making:** Building a culture of training in, and demand for, data-driven decision-making is crucial to improving data use and analytics within the health sector [Interview 4]. One method is to first conduct an analytic capacity needs assessment [Interview 4]. Based on the assessment, training programs aimed at developing advanced analytical skills could be designed. In addition to presenting or visualizing data effectively [Interviews 2, 4], these programs can focus on developing complex critical thinking skills [Interview 3], such as identifying relationships between factors [Interview 4], and enabling staff to perform more in-depth analyses than would have been possible with basic descriptive analyses. While Ghana has established a program to train personnel at lower levels of the health system in data analytics, expanding training to senior staff and directors, including within the MoH, would be beneficial [Interviews 3, 4] as this group more directly impacts the final stages of priority-setting.

Capacity improvement is needed not only in data analysis, but also in data generation. For example, to improve TB case detection, capacity improvement is needed among laboratory scientists, nurses and referral clinicians [Interview 8]. Another avenue to increase data proficiency across the health workforce could be task shifting; for example, a nurse learning how to input data so they can do so in the event of unavailability of a data manager [Interview 8]. Importantly, one interviewee also emphasized that expanding capacity building training programs alone is insufficient for staff to advance their analytic skills; programs must be complemented with a greater culture of demand for the use of advanced analytics to motivate staff, as well as to improve the attraction and retention of qualified statisticians from the academic or private sectors to the MoH [Interview 3].

1. **Establish centralized health research coordination agency:** The MoH should establish one national health research agency [Interview 3] to reduce fragmentation and duplication of efforts between the MoH’s Research, Statistics and Information Management Directorate and the GHS’ RDD, and to strengthen the coordination, performance, and capacity for health research in Ghana. Given that the MoH is the policy-making body, it would be beneficial to position this agency within the MoH [Interview 3].
2. **Integrate and coordinate health databases:** Currently, health data is not centralized within a single agency, making it imperative to enhance interoperability and integration within existing health information systems [9,34]. By establishing a national health database which integrates these systems, health data can be used more effectively, supporting a unified approach to HSPS [Interview 4].

## 5. Discussion

Using interviews with key informants and qualitative analysis, this study analyzed the role of data and analytics in Ghana’s HSPS. To establish the context of HSPS in Ghana, the paper presented a literature review of Ghana’s health system and HSPS processes. Then, data on interviews with key informants were presented to fill in gaps identified in the literature on the role of data and analytics in Ghana’s HSPS. Results from interviews show a low use of analytics for HSPS in Ghana. The paper concludes by offering recommendations, based on the interviews, to improve analytical capacity for HSPS.

Although the MoH leads in priority-setting, it relies mainly on assessments conducted by the GHS, its implementation agency. GHS assessments are mainly based on the DHIMS2 database and the analytic method used for an assessment depends on the purpose of the assessment. Methods used are mostly descriptive such as counts, averages and charts [Interviews 2, 4], though there is a desire to use more data visualization methods and advanced analytics [Interviews 2, 3, 4] such as cost-effectiveness analysis. The interviews revealed efforts being made to develop data and analytic capacity within the health sector [Interviews 2, 6, 7, 8], such as training of MoH representatives on the use of data for policy briefs and infographics [Interview 7]. However, many of these efforts are targeted at individuals who are not involved in priority-setting at the highest level, limiting their influence on the latter, yet critical stages of HSPS. Interviewees recommended to target such training at decision makers at the highest level including divisional directors [Interviews 3, 4]. The Individuals, Nodes, Networks, and Environment framework could serve as a valuable model for planning these capacity-building efforts. The framework emphasizes locally relevant technical skills in each stakeholder’s specific environment, maximizing the application of skills throughout the priority-setting processes [35].

An interviewee recommended the establishment of a centralized health research coordination agency as an important way to improve analytic capacity [Interview 3]. Indeed, integrating the country’s expertise, resources, and data into one health research agency is likely to help address challenges related to analytical capacity, human resources, absence of clear coordination mechanisms, and fragmentation in funding for health research. This action should also strengthen the use of data from in-country research to inform HSPS. Successful examples of national health research agencies exist in other African countries such as the National Institute for Medical Research in Tanzania, the Kenya Medical Research Institute and the South African Medical Research Council. Additional best practice examples on the use of public health data for decision making are demonstrated by the United States Centers for Disease Control and Prevention (CDC). For example, the CDC organizes influenza forecasting challenges in which external research groups use influenza hospitalizations and death data for previous seasons to predict future incidence to inform health resource planning [36]. Additionally, the Centers for Forecasting and Outbreak Analytics, an agency of US CDC focuses on producing cutting-edge models and forecasts “for public health decision makers to better prepare and respond to outbreaks” [37]. In these examples, the efficient use of data involves the use of modern analytic methods, collaboration between public health agencies and research institutions on data analysis and the production of analytic outputs that are relevant for decision making.

Like health research, health databases in Ghana are not connected or coordinated.

The DHIMS2 database used by the GHS for its assessments is separate from health databases used by teaching hospitals and some private health facilities and there is limited interoperability between these databases [9] [Interviews 2, 3]. A useful step towards achieving the recommendation of a national unified health database [Interview 4] would be to assess the technical readiness of each system for integration, data accessibility, data management and the relevance of indicators. Strengthening data reporting regulations, through HEFRA, to promote compliance is another needed action if systems integration is to be successful in practice. Ghana could also learn from countries with established national health databases such as in Sweden and Australia [38].

The ownership of national health databases is an important issue. To preserve patients’ privacy and to ensure the availability of critical public health data, any national database should be owned and managed by the government. When the software used to develop national health databases are proprietary and owned by entities other than the government, it makes the country vulnerable to decisions of owners to withhold data in the event of contractual misunderstandings. For example, an alleged delay in the Ghanaian government’s payment for the services of LHIMS, which is privately owned, has raised concerns about possible leaks of sensitive private health data as a bargaining tool [39]. Additionally, the recent cuts in USAID funding have led to restricted access to DHS data, limiting the opportunities for research and interventions on population health [40].

Despite the limited analytic capacity, analytical capacity does exist within the GHS [Interviews 2, 3, 4 and 5] and is used in generating evidence for health sector assessments. However, interviews showed that these assessments may not always be used in HSPS. Several interviewees suggested that prioritization in Ghana’s health sector is heavily influenced by the availability of resources and political influences rather than by objective criteria. This is likely to demotivate staff who have the capacity to perform advanced analytics, given that the analyses they conduct may not be considered in decision making. In addressing analytical capacity, it is therefore important to address other factors which may complement or compromise the role of data and analytics for HSPS.

One such factor noted by interviewees is donor priorities. However, there was a disagreement between interviewees on this point, with two development partner representatives—from WHO and PATH—claiming that their organizations’ activities align with government’s priorities [Interviews 1, 7]. It is noteworthy that there was no clear evidence of the use of criteria in the process of scoring priorities in the final business meeting (between government and donors) when health sector priorities are finalized. Therefore, decisions on priorities may likely not be backed by objective, data-based criteria.

Results from the literature support statements on donors’ influence on national priorities [41,42], despite attempts by donors themselves to develop aid mechanisms designed to promote enhanced country ownership in relation to donor-funded programs, such as the Ghanaian Country Coordinating Mechanism mandated by the Global Fund to Fight AIDS, Tuberculosis and Malaria [42]. Donor and political influences on HSPS could be ameliorated by establishing clear objective criteria for HSPS to help guide stakeholders and by empowering the public to contribute to setting the criteria while holding the government accountable to the set criteria. Committing to the generation of local funds for financing the implementation of priorities is a needed step if donor influences on health sector decisions are to be minimized.

Findings from the interviews corroborate with several results from the literature [43–46]. For example, a recently published systematic review on HTA studies conducted on health data from Ghana showed limited local capacity for HTA: only 30% of authors on these studies were Ghanaian, with Ghanaian author roles being data collection or study conceptualization and rarely data analysis [43]. This implies that though Ghanaian health service staff may collaborate on advanced analytics studies such as cost-effectiveness analysis for HTA, these staff do not take the lead on data analytics, likely because of their limited capacity in this area. Another study conducted on the state of health policy and system research in Ghana showed limited use of evidence in health decision making, citing reasons such as inadequate government funding for research and the lack of relevant engagement between decision makers and academic researchers [44].

While this study sought to address the gaps identified in the literature review on HSPS, this study was limited by its sampling strategy. Despite the small sample size, which is a limitation, it was determined that saturation was achieved by the eighth interview, providing assurance that key information regarding HSPS in Ghana had been sufficiently covered by the interviews. Also, interview responses were recorded by notetaking rather than verbatim transcription. While this approach allowed for efficient data collection, it may have resulted in the omission of certain details or nuances, which constitutes a limitation of the study. However, to minimize errors due to notetaking, notes were taken by two interviewers and were compared to achieve consistency. Again, despite efforts to ensure objectivity, researcher biases may have subtly influenced the interpretation and presentation of findings. Efforts have however been made to present findings transparently, with a ten-member research team informing analysis, interpretation and presentation of results over several iterations.

Despite these weaknesses, the study has several strengths, key of which is the use of both an in-depth literature review and interviews with key informants to present a broad and current view of HSPS in Ghana with a focus on the use of data and analytics. Another strength is the background and extensive experience of interviewees, which allowed the leveraging of institutional memory for the realization of useful insights, many of which were absent from the literature.

## Supporting information

S1 Text Further information from the literature on HSPS in Ghana. (DOCX)

S2 Text Interview guides. (DOCX)

S3 Text Interview notes. (DOCX)

S1 Checklist Inclusivity in global research. (DOCX)
